# Three-dimensional visualization of neural networks inside bone by Osteo-DISCO protocol and alteration of bone remodeling by surgical nerve ablation

**DOI:** 10.1038/s41598-023-30492-4

**Published:** 2023-03-22

**Authors:** Kurando Utagawa, Takaei Shin, Hironori Yamada, Hiroki Ochi, Satoko Sunamura, Aiko Unno, Chihiro Akazawa, Masatsugu Ema, Shu Takeda, Atsushi Okawa, Shingo Sato

**Affiliations:** 1 Department of Orthopaedic Surgery, Graduate School, Tokyo Medical and Dental University (TMDU), 1-5-45 Yushima, Bunkyo-Ku, Tokyo, 113-8519 Japan; 2 Faculty of Medicine, Tokyo Medical and Dental University (TMDU), Tokyo, 113-8519 Japan; 3 Department of Family Medicine, Graduate School, Tokyo Medical and Dental University (TMDU), Tokyo, 113-8519 Japan; 4 Japanese Red Cross Ishinomaki Hospital, Miyagi, 986-8522 Japan; 5 Department of Rehabilitation for Motor Functions, Research Institute, National Rehabilitation Center for Persons with Disabilities, Tokorozawa, Saitama 359-8555 Japan; 6 Intractable Disease Research Center, Juntendo University School of Medicine, Tokyo, 113-8421 Japan; 7 Department of Biochemistry and Biophysics, Graduate School of Medical and Dental Sciences, Tokyo Medical and Dental University (TMDU), Tokyo, 113-8510 Japan; 8 Department of Stem Cells and Human Disease Models, Research Center for Animal Life Science, Shiga University of Medical Science, Shiga, 520-2192 Japan; 9 Division of Endocrinology, Toranomon Hospital Endocrine Center, Tokyo, 105-8470 Japan; 10 Center for Innovative Cancer Treatment, Tokyo Medical and Dental University (TMDU), Tokyo, 113-8519 Japan

**Keywords:** Cell biology, Molecular biology, Molecular medicine

## Abstract

Bone is one of the largest organ systems in humans and is considered to regulate whole-body homeostasis in cooperation with other organs. We have previously reported that a sympathetic or sensory nervous system inside bone regulates bone homeostasis. However, the detailed regulatory mechanism, including the distribution of nerves inside bone, remains unknown. Although a two-dimensional histological analysis has been widely used to evaluate the structure of nerves or blood vessels, the actual structure is more complex, suggesting that it should be evaluated three-dimensionally. Here, we established a novel bone tissue clearing technique (Osteo-DISCO) for murine bones which enabled us to visualize the detailed distribution of nerves or blood vessels inside bone. Interestingly, we found that there is a specific nerve entry site in each long bone and that surgical ablation of the specific nerve fibers entering bone tissue led to decreased bone formation and impaired bone regeneration. Furthermore, we revealed that the administration of calcitonin gene-related peptide (CGRP), which is primarily released from sensory nerves, suppressed the bone loss caused by surgical nerve ablation. An in vitro study also indicated that CGRP directly promotes osteoblast activity, suggesting that sensory nerves inside bone can regulate osteogenesis via the secretion of CGRP.

## Introduction

Bone is one of the largest organ systems in humans, and bones account for 15% of human body weight and maintain the shape of the body in the presence of gravity. In addition, bone is considered an important component in the maintenance of whole-body homeostasis. Metabolic regulatory networks between bone and other organs, such as those regulating mineral homeostasis, glucose metabolism, energy metabolism and hematopoiesis, have been studied^1–3^, and some of these interactions are associated with the nervous system or neuropeptides^4,5^. Our group has previously reported that the sympathetic nervous system and neuropeptides regulate bone homeostasis^6–8^. Additionally, our group has also revealed that a sensory nerve system inside bone plays a crucial role in regulating bone remodeling^9^. Researchers currently postulate that sensory nerves and sympathetic nerves are widely distributed in the periosteum or bone marrow. Gao et al. recently demonstrated that sensory nerves are major nerve fibers in bone based on analyses of immunofluorescence staining^10^. However, the detailed neural distribution and neural network inside bone remain unknown.

In general, the peripheral nervous system forms three-dimensional (3D) networks. However, these structures have been widely evaluated by performing a two-dimensional (2D) histological analysis using frozen or paraffin sections. Only a portion of the nerve fibers are visible in the 2D analysis, and their 3D structure is difficult to understand. To overcome the weaknesses of 2D histological analysis, several clearing techniques for soft tissues have been recently reported^11–20^. Tissue clearing techniques are powerful tools for analyzing the 3D structures of fiber-like components (e.g., neurons and blood vessels). However, optical clearing of osseous tissues is quite challenging because bone tissue contains hydroxyapatite and type 1 collagen-rich properties, which prevent deep penetration of chemical reagents. In addition, bone tissue consists of a two-layer structure of cancellous bone and cortical bone, which makes it difficult to adjust the refractive index. Although a few bone clearing techniques (e.g., Cubic-R protocol for bone, PEGASOS, and vDISCO) have been reported thus far^21–23^, these protocols require a long time (a few weeks to a month) for bone tissue clearing. Aqueous reagents (e.g., amino alcohol N,N,N’,N’-tetrakis(2-hydroxypropyl)ethylenediamine) are good for maintaining fluorescence intensity, but a longer incubation time is needed compared with that needed for organic solvent-based tissue clearing methods. While organic solvent-based methods (e.g., using 1-methyl imidazole *tert-*butanol) achieve transparency compared to aqueous solvent-based methods, early fluorescence degeneration is inevitable. Thus, a modified protocol to clear the whole bone tissue in a shorter time while minimizing fluorescence degradation is needed.

In the current study, we established a new organic reagent-based bone clearing method (Osteo-DISCO) in which a shortened incubation time for clearing murine bones and the preservation of fluorescence intensity for more than 18 months were achieved. This method enabled us to observe the detailed 3D structure of the neurovascular networks inside bone. Additionally, we succeeded in identifying the specific nerve entry site in each murine long bone. Surgical nerve ablation at the nerve entry site of the murine tibia resulted in a decrease in the number of sensory nerves inside bone, which led to loss of bone mass and insufficient bone regeneration due to impaired bone formation. Furthermore, we also revealed that calcitonin gene-related peptide (CGRP), which is primarily released from sensory nerves, enhanced osteogenesis and suppressed the bone loss caused by surgical nerve ablation, suggesting that bone homeostasis can be regulated by sensory nerves inside bone.

## Results

### Development of a novel optical clearing method (Osteo-DISCO) for murine bones

**Figure 1:**
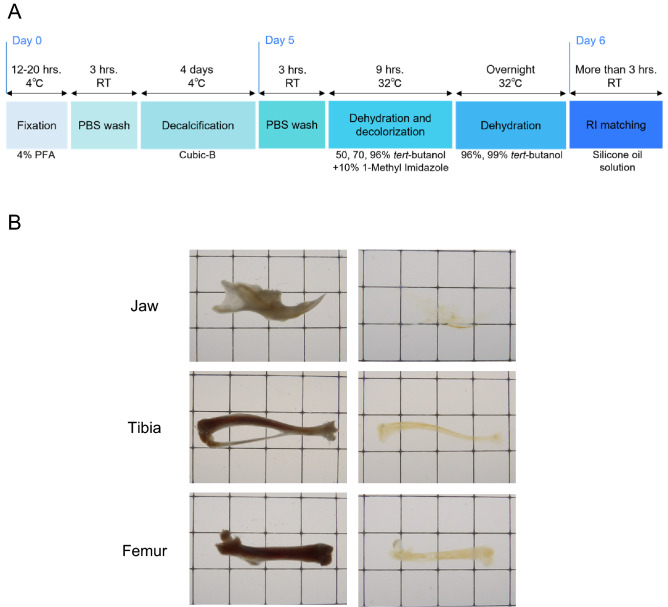
Establishment of a novel optical clearing method for murine bones (Osteo-DISCO). (**A**) Brief description of the new clearing procedure. (**B**) Murine bones (jaw, tibia, and femur) cleared using our procedure. Each image on the left side was captured before clearing, and images on the right side were captured after clearing. All bright field images were captured with bones immersed in silicone oil and on a 5 mm square of graph paper.

**Figure 2:**
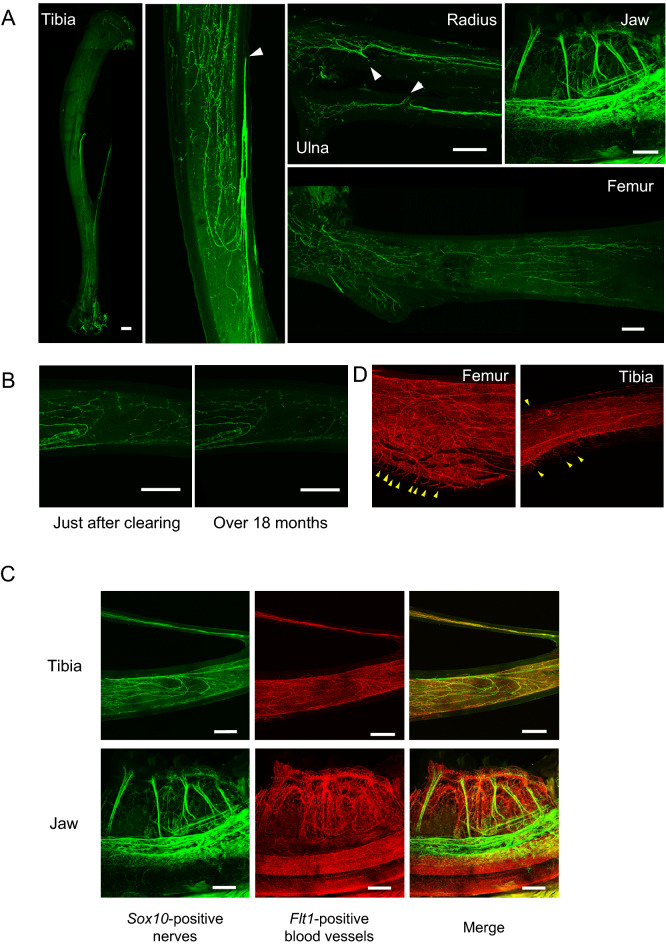
The newly established optical bone clearing (Osteo-DISCO) method reveals the 3D structure of the neurovascular network inside bone. (**A**) Visualized nerves inside bones of *Sox10-venus* mice (tibia: the whole and magnified midshaft, forearm: radius and ulna, jaw, and femur). White arrowhead: Specific entry site of *Sox10*-positive nerves. Scale bar: 500 µm. (**B**) Venus fluorescence of *Sox10*-positive nerves was preserved even 18 months after clearing. Scale bar: 500 µm. (**C**) Visualized nerves and blood vessels inside bones of *Sox10-Venus; Flt1-tdsRed* mice (tibia and jaw). Scale bar: 500 µm. (**D**) Blood vessels entering bone tissue in *Flt1-tdsRed* mice. Yellow arrowheads: entry sites of *Flt1*-positive blood vessels.

To visualize the internal structure of murine bones, we established a novel organic solvent-based bone clearing method, which is composed of fixation, decalcification, dehydration, delipidation, decolorization, refractive index (RI) matching and observation using confocal microscopy (Fig. 1A for the detailed protocol). This protocol was developed with reference to previous tissue clearing techniques. At the dehydration step, we tried an organic reagent, namely, a gradient of *tert-*butanol solutions, as described in previous studies^12,22^. In our protocol, 99% (v/v) *tert-*butanol was used at the final step of dehydration because compared with an incubation using 100% (v/v) *tert-*butanol, an incubation using 99% (v/v) *tert-*butanol prevented fluorescence quenching (data not shown). For the RI matching step, we selected a new RI matching solution based on silicone oil, which is composed of 72% (v/v) 1,3,3,5-tetramethyl-1,1,5,5-tetraphenyltrisiloxane and 28% (v/v) diphenylsiloxy phenyl trimethicone. Our RI matching solution achieved high transparency and good optical access throughout the whole murine bone, as well as long-term preservation of endogenous fluorescence for more than 18 months (Figs. 1B and 2B). Furthermore, our method enabled us to avoid additional dehydration or delipidation steps before RI matching, which were required in the previously reported tissue clearing protocols, reducing the processing time to 6 days at minimum, which is the shortest processing time for a tissue clearing protocol reported to date (Supplementary Fig. 1). Thus, we succeeded in developing a novel optical clearing method for murine bones with high transparency, longer-term fluorescence preservation and a shorter processing time and named our new procedure Osteo-DISCO.

### A newly established optical bone clearing method, Osteo-DISCO, reveals the 3D structure of the neurovascular network inside bone

To visualize the nerve distribution pattern inside murine bones, we used *Sox10-Venus* mice, in which mature and immature oligodendrocytes and Schwann cells are clearly labeled with Venus fluorescence^24^. Osteo-DISCO enabled us to observe the nerve distribution pattern in each murine bone, including the tibia, femur, ulna, radius and mandible (Fig. 2A). We also confirmed that Venus fluorescence of Sox10-positive nerves can be observed even 18 months after the bone clearing process (Fig. 2B). Interestingly, we identified a specific nerve entry site for each long bone (Fig. 2A, white arrowheads). After entering bone tissue, some nerve fibers extend to the proximal site, and others innervate the distal site. In the mandible and teeth, extensive branching of the inferior alveolar nerve was observed, in addition to vertical nerve fibers inside the dental pulp (Fig. 2A).

We also attempted to visualize the 3D vascular network inside murine bones. Flt-1, which is known as vascular endothelial growth factor receptor 1 (VEGFR-1), is widely expressed in vascular endothelial cells. Therefore, we used *Flt1-tdsRed* mice^25^ to visualize blood vessels inside bone and succeeded in observing numerous blood vessels using the same bone optical clearing method (Fig. 2C). Furthermore, by crossing *Sox10-Venus* mice with *Flt1-tdsRed* mice, we also successfully visualized both nerves and blood vessels at the same time (Fig. 2C and Supplementary Video 1). Interestingly, our observations confirmed that the distribution patterns of Flt1-positive blood vessels and Sox10-positive nerves were not necessarily the same. Some nerve fibers, especially thicker nerve fibers, extend alongside Flt1-positive blood vessels inside bone, but some thinner nerve fibers do not (Supplementary Fig. 2). According to previous studies, it has been reported that both nerves and blood vessels enter bone tissue at the same site^26^. However, we found that blood vessels enter bone tissue radially, not via a specific entry site. The entrance pattern of blood vessels was quite different from that of nerves in murine bones (Fig. 2D and Supplementary Fig. 2). Thus, our new optical clearing method, Osteo-DISCO, is a useful tool for analyzing fiber-like structures such as nerves and blood vessels three-dimensionally.

### Surgical nerve ablation at the nerve entry site induces bone loss via reduced bone formation

**Figure 3:**
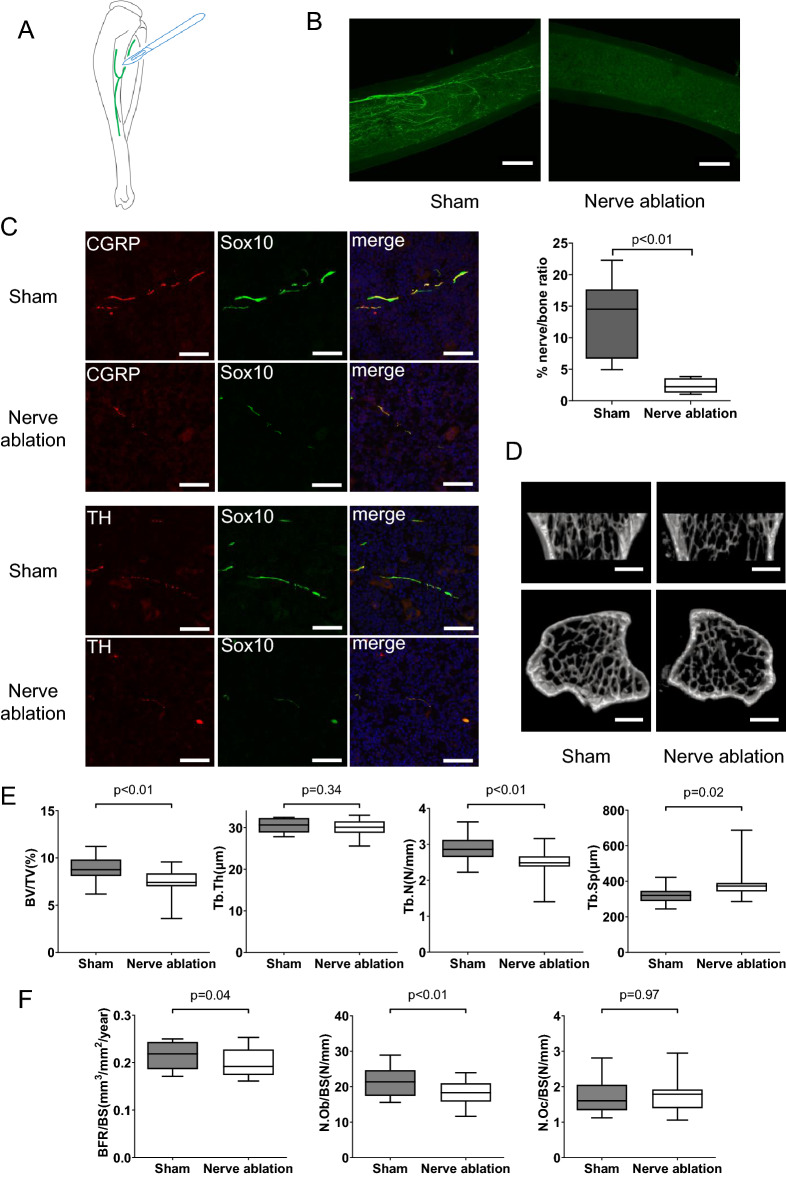
Surgical nerve ablation at the nerve entry site induces bone loss via reduced bone formation. (**A**) Schematic diagram of surgical nerve ablation. Nerve fibers entering the tibia were surgically ablated at the entry site. (**B**) Representative Osteo-DISCO images of *Sox10*-positive nerves inside the tibia in the sham operation group and nerve ablation group (upper) and % nerve/bone ratio: percentage of nerve fibers in tibia bone area on the Z-stack image (lower). The number of *Sox10*-positive nerves was remarkably reduced in the nerve ablation group. Scale bars: 500 µm. These data are presented as the means ± SEMs (n = 6). P values were obtained using a two-tailed paired t test. (**C**) Representative images of immunofluorescence staining for CGRP, TH, in both groups. Scale bars: 50 µm. CGRP: calcitonin gene-related peptide, TH: tyrosine hydroxylase, EMCN: endomucin. (**D**) Micro-CT images of proximal tibiae in both groups showing the decrease in bone mass in the nerve ablation group. (**E**) Micro-CT analysis of trabecular bone in proximal tibiae showing a significant decrease in bone volume and trabecular number in the nerve ablation group. BV/TV: bone volume/tissue volume, Tb.Th: trabecular thickness, Tb.N: trabecular number, Tb.Sp: trabecular separation. (**F**) Bone histomorphometric analysis of trabecular bone in proximal tibiae showing reduced bone formation in the nerve ablation group. BFR/BS: bone formation rate/bone surface, N.Ob/BS: osteoblast number/bone surface, Ob.S/BS: osteoblast surface/bone surface, N.Oc/BS: osteoclast number/bone surface, Oc.S/BS: osteoclast surface/bone surface. All data are presented as the means ± SEMs (n = 10). P values were obtained using a two-tailed paired t test (**E** and **F**).

We identified a specific nerve entry site for each long bone, as described above. To verify the role of the entering nerves in bone homeostasis, we established a new surgical nerve ablation model. According to our observation of the cleared tibiae of *Sox10-Venus* mice, the nerve entry site is located at the posterior surface of the middle to proximal tibia. We ablated the entering nerves at the entry site, as shown in Fig. 3A. The surrounding periosteum and muscles were partially damaged by this procedure. Therefore, to eliminate the influence of the damaged tissues, we conducted the same procedures at the lateral surface of the contralateral tibia without ablating nerves as a sham operation. Optical clearing of tibiae collected from the nerve-ablated *Sox10-Venus* mice showed that compared with sham-operated tibiae, tibiae subjected to surgical nerve ablation exhibited remarkably reduced nerve fibers inside the tibiae after two weeks (Fig. 3B). In addition, we performed immunohistochemical (IHC) staining of tibiae collected from nerve-ablated *Sox10-Venus* mice to evaluate the effect of nerve ablation on the neurovascular network inside the tibiae. In the sham operation group, we found that Sox10-positive nerve fibers coexpressed CGRP, which is a marker of sensory nerves, or tyrosine hydroxylase (TH), which is a marker of adrenergic sympathetic nerves (Fig. 3C), and that CGRP-positive sensory nerves were more predominant inside bone, as reported in previous studies^10^. We also confirmed that the numbers of both CGRP-positive nerves and TH-positive nerves were noticeably decreased in the nerve-ablated tibiae compared with the sham-operated tibiae (Fig. 3C), suggesting that most nerves inside bone were derived from the nerves entering via a specific entry site. In addition, IHC staining using an anti-endomucin (EMCN) antibody and anti-CD31 antibody showed that the volume of EMCN-positive venous sinusoids and CD31-positive arterial capillaries inside bone was not changed after surgical nerve ablation (Supplementary Fig. 3) because the blood circulation inside bone is provided by many transcortical capillaries. We also confirmed that the volume of Type H vessels with high expression of both EMCN and CD31, which are mainly distributed on the endocortical surface and have the ability to induce bone formation, was also unchanged between the sham and denervation groups (Supplementary Fig. 3). Thus, these findings suggest that our nerve ablation procedure affects only the nerves inside bone.

Furthermore, to evaluate the effect of nerve ablation on bone remodeling, we performed microcomputed tomography (micro-CT) analysis and bone histomorphometry analysis using tibiae collected 2 or 4 weeks after the nerve ablation procedure. The micro-CT analysis revealed that nerve ablation significantly decreased the bone volume/tissue volume (BV/TV) and trabecular number (Tb.N) and increased trabecular separation (Tb.Sp) in the proximal tibiae (Fig. 3D,E). The bone histomorphometric analysis revealed that nerve ablation significantly decreased the bone formation rate (BFR/BS), osteoblast number (N.Ob/BS) and osteoblast surface (Ob.S/BS), whereas there was no significant change in the osteoclast number (N.Oc/BS) or osteoclast surface (Oc.S/BS) (Fig. 3F). Based on these results, surgical nerve ablation at the nerve entry site mainly reduces the number of CGRP-positive sensory nerves inside the tibiae, resulting in bone loss via decreased bone formation without affecting bone resorption.

### Surgical nerve ablation also suppresses bone regeneration

**Figure 4:**
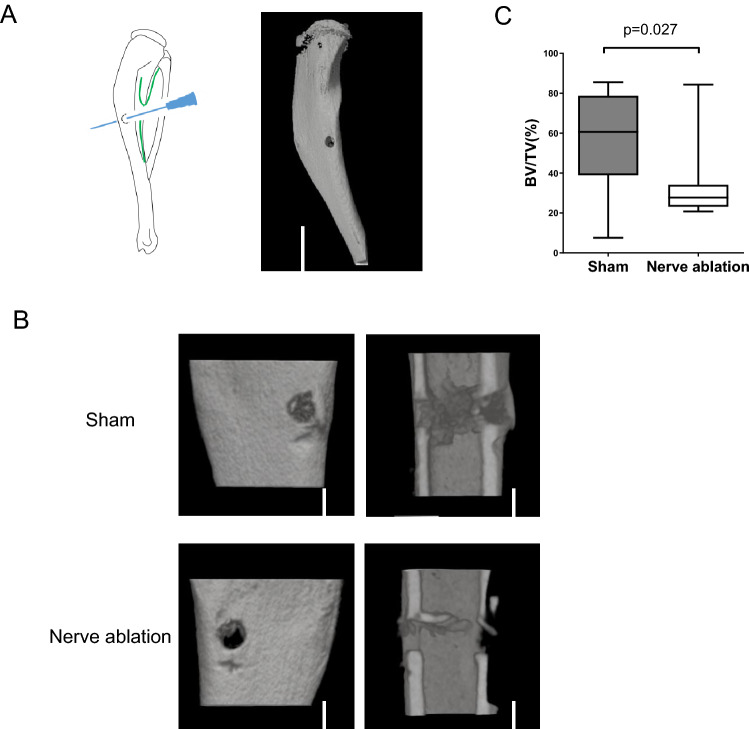
Surgical nerve ablation also suppresses bone regeneration. (**A**) Schematic diagram of the procedure used to create the drill hole model (left panel). A micro-CT image captured after the creation of drill holes (right panel). Scale bar: 3000 µm. (**B**) Micro-CT 3D reconstruction images and coronal planes of the drilling site in the sham operation group and nerve ablation group, showing impaired bone regeneration in the nerve ablation group. Scale bars: 600 µm. (**C**) Micro-CT analysis of the regenerated cortical bone mass at the drilling site. All data are presented as the means ± SEMs (n = 4). P values were obtained using a two-tailed paired t test (**C**).

We also hypothesized that nerves inside bone play an important role in bone regeneration. To evaluate the influence of nerve ablation on bone regeneration, we established a drill hole model, as shown in Fig. 4A. We drilled bicortical holes using a 27 G syringe needle at the middle of murine tibiae, avoiding the nerve entry site. Nine days after the drilling procedure, the drilled tibiae were harvested, and the volume of the regenerated cortical bone mass at each drilling site was measured using micro-CT. The micro-CT analysis showed that bone regeneration was significantly impaired in the nerve-ablated tibiae (Fig. 4B,C), suggesting that nerves inside bone are also critical for bone regeneration.

### CGRP administration rescues the bone loss induced by surgical nerve ablation

**Figure 5:**
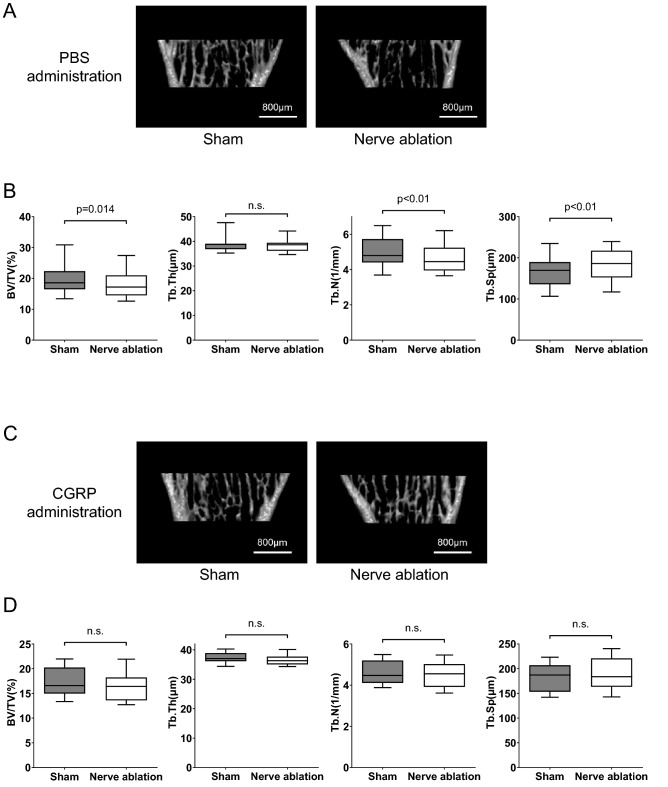
CGRP administration rescues the bone loss induced by surgical nerve ablation. (**A**) Representative micro-CT images of proximal tibiae in the sham operation group and nerve ablation group treated with PBS. Scale bars: 800 µm. (**B**) Micro-CT analysis of trabecular bone in proximal tibiae, showing a significant decrease in bone mass and trabecular number in the nerve ablation group treated with PBS. (**C**) Representative micro-CT images of proximal tibiae in both groups treated with CGRP. Scale bars: 800 µm. (**D**) Micro CT analysis of trabecular bone in proximal tibiae, showing that the bone loss caused by nerve ablation was rescued by CGRP administration. All data are presented as the means ± SEMs (n = 15). n.s.: not significant. P values were obtained using a two-tailed paired t test (**B** and** D**).

Based on the aforementioned findings, we hypothesized that CGRP-positive sensory nerves inside bone are important for maintaining bone homeostasis. To verify this hypothesis, we performed continuous administration of CGRP or PBS for 4 weeks using an osmotic pump in wild-type mice subjected to surgical nerve ablation. An osmotic pump was implanted into the subcutaneous space of each mouse. Nerve ablation and the sham operation were performed in the right and left tibia of each mouse, respectively, before administration. In the PBS administration group, surgical nerve ablation significantly reduced bone mass (BV/TV) and trabecular number (Tb.N) compared with the sham procedure (Fig. 5A,B). On the other hand, we did not identify any significant differences in any parameter between the nerve ablation side and the sham operation side in the CGRP administration group (Fig. 5C,D). Thus, bone loss induced by surgical nerve ablation is substantially rescued by continuous CGRP treatment, suggesting the possibility that CGRP secreted by sensory nerves inside bone contributes to bone remodeling.

### CGRP directly promotes osteogenesis

**Figure 6:**
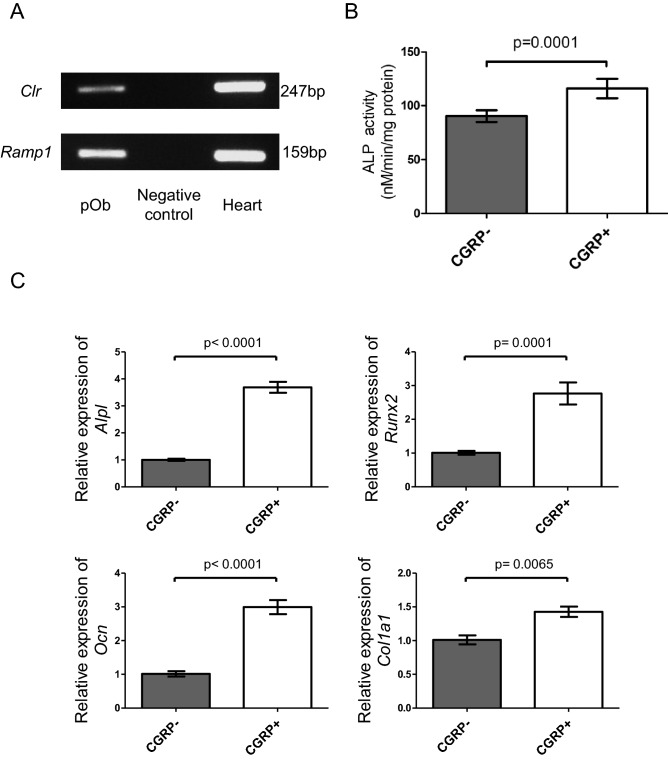
CGRP directly promotes osteogenesis in vitro. (**A**) Gene expression of two subunits of the CGRP receptor (*Clr* and *Ramp1*) in osteoblasts detected using PCR. Clr: calcitonin gene-related peptide type 1 receptor, Ramp1: receptor activity modifying protein 1. The negative PCR control contained no RNA. Murine heart samples were used as a positive control. (**B**) ALP activity was increased in CGRP-treated primary murine osteoblasts after 4 days of osteogenic induction. (**C**) qPCR analysis showing higher expression of osteogenic genes (*Alpl*, *Runx2*, *Ocn*, and *Col1a1*) in CGRP-treated primary murine osteoblasts after 2 days of osteogenic induction. *Gapdh* was used as an internal control. All data are presented as the means ± SEMs (n = 6). P values were obtained using Student’s t test (**B** and **C**).

The functional CGRP receptor is composed of calcitonin receptor-like receptor (CLR) and receptor activity-modifying protein 1 (RAMP1)^27^. We confirmed that both genes were expressed on primary osteoblasts (Fig. 6A). To assess the effect of CGRP on osteoblast activity and differentiation, we added a physiological amount (10^−12^ M) of CGRP to the culture medium of primary osteoblasts during osteoblast differentiation. After a 4-day osteogenic induction period, an alkaline phosphatase assay revealed that alkaline phosphatase activity was increased by the addition of CGRP. Quantitative real-time PCR also showed that the gene expression levels of osteogenic markers such as *Alpl*, *Runx2*, *Ocn* and *Col1a1* were upregulated in the CGRP-treated osteoblasts after 2 days of osteogenic induction.

## Discussion

Many studies investigating the neural regulation of bone remodeling have been reported to date. In previous studies, our group documented that a sympathetic nervous system innervates bone and that an increase in sympathetic nervous activity induces bone loss via β2-adrenergic receptor signaling in osteoblasts^6^. A recent study by Sundeep Khosla et al. reported that sympathetic β1-adrenergic signaling also contributes to the regulation of human bone metabolism^28^. In animal experiments, β-blocker administration increased bone mass and improved biomechanical fragility^6^. Epidemiological studies have also shown a 15–30% lower risk of fracture in patients receiving β-blockers^29–31^. However, by performing a detailed analyses of mice lacking Sema3A in neurons (*Sema3a*^*synapsin−/−*^ and *Sema3a*^*nestin−/−*^ mice), our group revealed that sensory nerve innervation into bone is essential for maintaining bone mass^9^. Subsequently, several animal studies by other researchers have also shown that the chemical or surgical denervation of sensory nerves leads to significant bone loss^32–34^. These studies suggest that both sympathetic and sensory nerves are important for regulating bone homeostasis. However, the detailed neural distribution inside bone remains unclear because visualizing the whole 3D structure of the neural network inside bone is challenging.

Several useful tissue clearing techniques have been developed and allowed us to visualize 3D neurovascular networks in a variety of murine soft tissues, such as the brain, peripheral nerve, kidney, heart and lung^13,35^. A few bone clearing techniques have also been published to date, but these protocols require a longer processing time to clear bone, and the visualization of nerves inside bone is insufficient. By selecting the best organic reagents and a new silicone oil-based RI matching solution, we modified the previous organic solvent-based clearing methods and successfully established a novel bone clearing technique, Osteo-DISCO, with high transparency, a shorter processing time (6 days at minimum) and longer preservation of fluorescence intensity (Fig. 1A).

Generally, long-term immersion in organic reagents is required for better clearing. However, organic reagents, including *tert-*butanol, easily decrease endogenous fluorescence. We found that compared with an incubation using 100% (v/v) *tert-*butanol, an incubation using 99% (v/v) *tert-*butanol in the final step of dehydration prevented the disappearance of fluorescence in our study (data not shown). Thus, this approach overcomes the fundamental problem that long-term immersion in organic reagents leads to degradation of fluorescence, and it has also been suggested that fluorescence degradation caused by organic reagents may be rescued by adding a small amount of water. For the RI matching step, we selected a new silicone oil-based RI matching solution. Silicone oil is a nontoxic polymer known for its great chemical stability and biological safety. Considering its characteristics, we hypothesized that the silicone oil solution might prevent early loss of endogenous fluorescence, which has been reported in previous organic reagent-based tissue clearing protocols. As expected, our RI matching solution achieved high transparency and good optical access throughout the whole murine bone, as well as long-term preservation of endogenous fluorescence for more than 18 months (Figs. 1B and 2B).

Using Osteo-DISCO, we were able to clearly observe the distribution pattern of nerves in murine bones such as the tibia, radius, ulna and mandible (Fig. 2A). However, it is still difficult to achieve sufficient optical access in thicker bones or thicker sites with a complicated shape, such as the femur, vertebra or epiphysis of the long bones. Thicker osseous sites contain more hydroxyapatite and are richer in type 1 collagen, which prevents reagents from penetrating deeply. We are working on further improvements for clearing thicker bones.

Through observations using Osteo-DISCO, we successfully identified a specific nerve entry site in each murine long bone, such as the tibia, radius and ulna (Fig. 2A). Thus far, researchers have postulated that nerves innervate bone accompanied by blood vessels via numerous entry sites in the periosteum^26^. However, interestingly, the current study indicated that the entry pattern of nerves was quite different from that of blood vessels in murine bones (Fig. 2A,D).

Based on the findings described above, we established a novel surgical nerve ablation model, as shown in Fig. 3A. The periosteum on the posteromedial edge of the tibial shaft, which is equivalent to the specific nerve entry site, was surgically scraped to eliminate the nerves entering the tibia. To reveal the relationship between the nervous system and bone homeostasis, the following three types of nerve ablation models have been reported to date. (1) Nordsletten et al. and Madsen et al. showed that surgical ablation of a sciatic nerve and/or a femoral nerve caused delayed fracture healing^33,34^. (2) Apel et al. and Ding et al. showed that chemical sensory nerve ablation via the injection of a high dose of capsaicin, which is a ligand for transient receptor potential cation channel subfamily V member 1 (TRPV1), induced bone loss and insufficient fracture healing^36,37^. (3) Hao Chen et al. generated a sensory denervation mouse model, *TrkA*_*Avil*_^*−/−*^ mice, by crossing sensory nerve-specific cre (*Advillin-cre*) mice with tropomyosin receptor kinase A (TrkA) floxed mice and observed that CGRP-positive sensory nerve fibers were eliminated and significant bone loss occurred in the femur and vertebra of *TrkA*_*Avil*_^*−/−*^ mice^5^. However, each previous denervation model has advantages and disadvantages. (1) Sciatic or femoral nerve ablation is very simple and easy to perform, but it also affects muscles innervated by the nerve and causes a decrease in muscle strength and physical activity. (2) Chemical denervation induced by capsaicin is not specific to the sensory nerve. Capsaicin acts as an excitatory neurotoxin and induces the overactivation of TRPV1 on sensory nerve fibers. However, TRPV1 is also expressed in chondrocytes, osteoblasts, osteoclasts, and osteoclast precursors^38,39^. (3) Genetic denervation using sensory nerve-specific knockout mice is more useful and reliable. However, it takes a long time to generate genetically modified mice. In addition, chemical and genetic denervation are not able to exclusively eliminate nerves in a specific bone. Our new surgical nerve ablation model eliminated only nerves entering the tibia without affecting the surrounding muscles or other organs and reduced nerve innervation inside the tibia as well as a decrease in bone mass and impaired bone regeneration. Orthopedic surgeons sometimes treat tibial shaft fractures. Interestingly, it is known that delayed union is a common complication of tibial shaft fractures^40^. It is possible that traumatic nerve ablation accompanied by bone fracture might be associated with the delay of fracture healing.

It is also known that both sympathetic nerves and sensory nerves exist inside bone. IHC staining confirmed that Sox10-positive nerve fibers inside bone coexpressed CGRP or TH (Fig. 3C) and that CGRP-positive sensory nerves were more predominant inside bone. A recent study by Gao et al. also revealed that approximately 77% of nerves inside bone are positive for CGRP^10^, indicating that the vast majority of nerves inside bone are sensory nerves. Our surgical nerve ablation technique resulted in both sensory and sympathetic denervation inside the tibia (Fig. 3C) and induced bone loss due to decreased bone formation (Fig. 3D–F). According to previous studies, the inhibition of sympathetic nervous activity induces an increase in bone mass^6^. Nonetheless, our nerve ablation technique decreased bone mass, suggesting that a decrease in the number of sensory nerves substantially contributes to the change in bone mass. Indeed, systemic continuous administration of CGRP to mice subjected to surgical nerve ablation successfully rescued the bone loss caused by nerve ablation (Fig. 5C,D). These findings indicate that CGRP, which is mainly secreted by sensory nerves, is one of the mediators that regulates bone remodeling in vivo.

We also confirmed that primary osteoblasts express a functional CGRP receptor and that CGRP directly promotes osteoblast activity and osteoblast differentiation in vitro (Fig. 6A–C). Previous studies have also demonstrated that CGRP increases the proliferation of osteoprogenitors and upregulates osteogenic gene expression^41,42^. Furthermore, Yifeng Zhang et al. revealed that CGRP promotes osteogenic differentiation via cAMP/PKA signaling through the phosphorylation of CREB1^43^. According to Ri Zhou et al., CGRP promotes the expression of osteoblast-related genes such as *Runx2*, *Ocn* and *Col1a1* through the activation of canonical Wnt/beta-catenin signaling^44^. These findings indicate that CGRP secreted by sensory nerves directly acts on osteoblasts and upregulates osteogenic genes through downstream signaling.

In conclusion, we established a novel bone clearing method, Osteo-DISCO, which enabled us to visualize the detailed 3D distribution of neurovascular networks inside murine bones. Osteo-DISCO can be extremely useful for elucidating the pathophysiology of various bone diseases, such as osteoporosis, fracture healing and osteoarthritis. Additionally, our original surgical nerve ablation method induced a decrease in the number of CGRP-expressing sensory nerves inside bone, resulting in loss of bone mass and insufficient bone regeneration due to impaired bone formation. Furthermore, CGRP enhanced osteogenesis in vitro and reversed the bone loss caused by surgical nerve ablation in vivo. These findings suggest that CGRP-expressing sensory nerves inside bone play a very important role in regulating bone remodeling.

## Materials and methods

### Mice

*C57BL/6* mice were purchased from the Jackson Laboratory. *Sox10-Venus* mice were provided by Chihiro Akazawa (Juntendo University School of Medicine), and *Flt1-tdsRed* mice were provided by Masatsugu Ema (Shiga University of Medical Science). Male mice were used in all animal experiments. All animal experiments were conducted with approval of the Animal Study Committee of Tokyo Medical and Dental University (#A2022-127A) and performed in accordance with the ARRIVE guidelines. All experimental methods conformed to the relevant guidelines and laws.

### Preparation of bone clearing reagents

Cubic-B: As described before, Cubic-B reagent was prepared by mixing 10% (w/v) ethylenediaminetetraacetic acid (EDTA) and 15% (w/v) imidazole in distilled water. The solution was constantly stirred at room temperature until the EDTA and imidazole dissolved completely. Cubic-B reagent was stored at 4 ℃.

Dehydration solutions: To prepare the dehydration solutions, 1-methyl imidazole was added to each 50% (v/v), 70% (v/v), and 96% (v/v) *tert-*butanol in distilled water up to a final concentration of 10% (v/v). The final concentrations of *tert-*butanol were 45%, 63%, and 86.4%, respectively. Ninety-six percent (v/v) and 99% (v/v) *tert-*butanol in distilled water without 1-methyl imidazole were used for the final steps of dehydration. All procedures were performed under a fume hood to prevent inhalation. As the freezing point of *tert-*butanol is 25–26 ℃, it must be maintained above room temperature. The dehydration solutions were stored at room temperature.

In order that the refractive index (RI) of the silicone oil solution matched that of a bone, A silicone oil solution was prepared by mixing 72% (v/v) 1,3,3,5-tetramethyl-1,1,5,5-tetraphenyltrisiloxane (HIVAC-F4; Shin-Etsu Chemical, RI = 1.555) and 28% (v/v) diphenylsiloxy phenyl trimethicone (KF-56A; Shin-Etsu Chemical, RI = 1.498).

### Optical bone clearing procedures for murine bones: Osteo-DISCO

Mice were deeply anesthetized by administering an intraperitoneal injection of a mixture of medetomidine (0.3 mg/kg), midazolam (4.0 mg/kg) and butorphanol (5.0 mg/kg). One hundred milliliters of heparin in PBS (10 U/ml heparin in 0.01 M PBS) was injected transcardially to remove blood, followed by 50 ml of 4% paraformaldehyde (PFA) for fixation. Each murine bone was carefully harvested, and the surrounding muscles or other tissues were removed. Bones were fixed with 4% PFA again with gentle shaking at 4 ℃ for 12–20 h. All the procedures described below were performed under gentle shaking. To remove PFA completely, the bones were washed with PBS for 1 h three times at room temperature. For decalcification, the bones were immersed in Cubic-B reagent for 4–7 days at 4 ℃ with a daily reagent exchange. The bones were washed again with PBS for 30 min to 1 h three times at room temperature. For decolorization and dehydration, the bones were sequentially immersed into 50% (v/v), 70% (v/v) and 96% (v/v) dehydration solutions containing 1-methylimidazole, as described above, at 32 ℃ for 3 h each. Next, the bones were immersed in 96% (v/v) *tert-*butanol at 32 ℃ for 12–16 h. In the final dehydration step, the bones were immersed in 99% (v/v) *tert-*butanol at 32 ℃ for 1–3 h. Finally, the bones were optically cleared by immersion in the silicone oil solution at room temperature for at least 3 h. The fluorescence of cleared bones can be preserved in silicone oil solution at room temperature in the light shade for more than a year. The neurovascular networks inside the cleared bones were examined using confocal microscopy (A1R; Nikon) and analyzed using ImageJ software.

### Animal experiments

Surgical nerve ablation model: Eight-week-old mice were used after deep anesthetization, as described above. Surgical nerve ablation and sham procedures were performed on the left and right hindlimbs of the same mouse, respectively. For surgical nerve ablation, a longitudinal skin incision was made on the anteromedial side of the left hindlimb. The periosteum on the posteromedial edge of the tibial shaft was surgically scraped through the space between the tibia and muscles. For sham surgery, a skin incision was made on the anterolateral side of the right hindlimb. The periosteum on the posterolateral edge of the tibial shaft was surgically scraped, similar to the nerve ablation procedure. After each skin incision was sutured, the mouse received an intraperitoneal injection of atipamezole (1.0 mg/kg) for the reversal of sedation. Ten pairs of tibiae were harvested 2 or 4 weeks after the nerve ablation procedure for further experiments.

Continuous administration of CGRP: To administer CGRP continuously, an osmotic pump (ALZET) was placed in the dorsal subcutaneous space. CGRP (4163-V; Peptide Institute, Inc.) was continuously administered at a dose of 2.4 μg/day for 4 weeks. In this experiment, CGRP-administered mice (n = 14) and PBS-administered mice (n = 15) were analyzed.

Drill hole model: Bicortical holes were created by drilling with a 410 µm (27 G) syringe needle from the medial border of the tibia to the lateral side. The drilled tibiae were harvested 9 days after the drilling procedure for further experiments. In this experiment, 5 mice were analyzed.

### Micro-CT analysis

The X-ray micro-CT analysis of murine tibiae was performed using ScanXmateL090 (Comscantecno). The images were analyzed using TRI/FCS-BON (Ratoc System Engineering) and the following parameters: X-ray tube potential: 90 kVp, X-ray intensity: 48 µA, number of projections: 1200, integration time: 5.0 f/sec, Shepp-Logan filter, 992 slices on each axis, and resolution: 11.360 µm on each axis.

### Histology and immunohistochemistry

For the immunohistochemical analysis, tibiae were fixed with 4% PFA for 4 h. Following 3 PBS washes for 30 min, the bones were incubated with decalcification buffer (20% EDTA, pH adjusted to 8.0) for 48 h. The samples were embedded in 4% carboxymethyl cellulose (CMC), snap-frozen in liquid nitrogen, and sliced into 8 µm sections using a cryostat (CryoStar NX70, Thermo Fisher Scientific Inc.). The sections were incubated with the following primary monoclonal antibodies for 16 h at 4 ℃: anti-CGRP antibody (#T4238, BMA Biomedicals), anti-TH antibody (#AB152, Sigma–Aldrich), anti-EMCN antibody (#sc-65495, Santa Cruz Biotechnology), and anti-CD31 antibody (AF3628, R&D Systems). 4',6-Diamidino-2-phenylindole (DAPI) was used to stain nuclei. The sections were observed using a confocal microscope (A1R; Nikon).

For bone histomorphometry, we injected mice with calcein (25 mg/kg, Sigma) intraperitoneally 5 and 2 days before sacrifice. After murine tibiae were harvested, the tibiae were embedded in methyl methacrylate resin, and the resin blocks were sliced to a 5-μm thickness. The undecalcified sections of the tibiae were stained with Villanueva bone staining. All bone histomorphometric parameters were measured at the secondary spongiosa region. To exclude the primary spongiosa, the measurement region was 0.23–1.40 mm distal to the lowest point of the growth plate. Static and dynamic histomorphometric analyses were performed using the Histometry RT CAMERA (System Supply, Nagano, Japan) at × 400 magnification at Niigata Bone Science Institute (Kizaki 761, Kita-Ku, Niigata city, Niigata, Japan). Results are reported as parameters recommended by the American Society for Bone and Mineral Research. The statistical analysis was performed using a two-tailed paired t test. In this histomorphometric analysis, 10 twelve-week-old male mice with unilateral nerve ablation were analyzed.

### Cell culture

In vitro primary osteoblast cultures were established as previously described^7^. Briefly, calvarial osteoblastic cells were isolated from 4-day-old mice by enzymatic digestion in α-minimal essential medium (α-MEM) with 0.5 mg/ml collagenase-P (Roche) and 0.05% trypsin. For serum starvation, cells (2 × 10^4^ cells per cm^2^) were cultured in Opti-MEM (Gibco) for 3 days. Then, the medium was changed to osteogenic medium (0.1 mg/ml ascorbic acid and 10 mM β-glycerophosphate) to induce osteoblast differentiation. The medium was changed on days 1 and 3. For CGRP treatment, CGRP was added to the osteogenic medium at a final concentration of 10^−12^ M. Cells were collected on day 2 for quantitative RT–PCR analysis and on day 4 for the alkaline phosphatase activity assay. Alkaline phosphatase activity was measured by using an ALP assay kit (291-58601, Wako).

### Quantitative RT–PCR analysis

Total RNA was extracted from cultured cells using TRIzol reagent (Invitrogen), and reverse transcription was performed with a ReverTra Ace qPCR RT Kit (TOYOBO) according to the manufacturer’s instructions. We performed a quantitative analysis of gene expression using the Mx3000P real-time PCR system (Agilent Technologies). *Gapdh* expression served as an internal control.

The following primers were used:

*Alpl* sense, 5′-ACACCTTGACTGTGGTTACTGCTGA-3′ and *Alpl* antisense, 5′-CCTTGTAGCCAGGCCCGTTA-3′; *Gapdh* sense, 5′-ACCCAGAAGACTGTGGATGG-3′ and *Gapdh* antisense, 5′-CACATTGGGGGTAGGAACAC-3′; *Runx2* sense, 5’- GCCGGGAATGATGAGAACTA -3’ *Runx2* antisense, 5’- ATGCGCCCTAAATCACTGAG -3’; *Ocn* sense, 5’- TCTGACAAAGCCTTCATGTCCA-3’*, Ocn* antisense, 5’- CGGTCTTCAAGCCATACTGGTC-3’; and *Col1a1* sense, 5′-ACGTCCTGGTGAAGTTGGTC-3′ and *Col1a1* antisense, 5′-CAGGGAAGCCTCTTTCTCCT-3′.

### Statistical analysis

Statistical analyses of all animal experiments were performed using a two-tailed paired t test. Statistical analyses of the RT‒PCR and ALP results were performed using a two-tailed Student’s t test, and we confirmed that each dataset followed a normal distribution using the Shapiro‒Wilk test.

Differences were considered significant at *P* < 0.05. All of the data are presented as the means ± standard errors of the means (SEMs). The results are representative of more than three individual experiments.

## Supplementary Information

Supplementary Information 1.

Supplementary Video 1.

## Data Availability

Data generated or analyzed during this study are included in the manuscript.
