# Supplementary material for: Three-dimensional visualization of neural networks inside bone by Osteo-DISCO protocol and alteration of bone remodeling by surgical nerve ablation
Source: Sci Rep. 2023 Mar 22;13:4674. doi: 10.1038/s41598-023-30492-4 (PMC10033912; doi:10.1038/s41598-023-30492-4)
Supplement: Supplementary file 1 — Supplementary Information 1. [file 41598_2023_30492_MOESM1_ESM.pdf]

Three-dimensional visualization of neural networks inside bone by Osteo-DISCO protocol and alteration of bone remodeling by surgical nerve ablation

<Authors>

Kurando Utagawa[1*], Takaei Shin[2,4*], Hironori Yamada[3*], Hiroki Ochi[5], Satoko Sunamura[1], Aiko Unno[1], Chihiro Akazawa[6,7], Masatsugu Ema[8], Shu Takeda[9], Atsushi Okawa[1], Shingo Sato[1,10,11]

*These three authors contributed equally to this work.

1. Department of Orthopaedic Surgery, Tokyo Medical and Dental University (TMDU), Graduate School, Tokyo 113-8519, Japan

2.Faculty of Medicine, Tokyo Medical and Dental University (TMDU), Tokyo 113-8519, Japan

3. Department of Family Medicine, Tokyo Medical and Dental University (TMDU), Graduate School, Tokyo 113-8519, Japan

4. Japanese Red Cross Ishinomaki Hospital, Miyagi 986-8522, Japan

5. Department of Rehabilitation for Motor Functions, Research Institute, National Rehabilitation Center for Persons with Disabilities, Tokorozawa, Saitama 359-8555, Japan

6. Intractable Disease Research Center, Juntendo University School of Medicine, Tokyo 113-8421, Japan

7. Department of Biochemistry and Biophysics, Graduate School of Medical and Dental Sciences, Tokyo Medical and Dental University (TMDU), Tokyo 113-8510, Japan

8. Department of Stem Cells and Human Disease Models, Research Center for Animal Life Science, Shiga University of Medical Science, Shiga 520-2192, Japan

9. Division of Endocrinology, Toranomon Hospital Endocrine Center, Tokyo 105-8470, Japan

10. Center for Innovative Cancer Treatment, Tokyo Medical and Dental University (TMDU), Tokyo, 113-8519, Japan

11. To whom correspondence should be addressed.1-5-45 Yushima, Bunkyo-ku, Tokyo 113-8519, Japan Phone: +81-3-5803-4865 E-mail: satoshin.phy2@tmd.ac.jp (Shingo Sato)

Supplementary Figure 1

	Osteo-DISCO	PEGASOS	vDISCO	Cubic-R	Bone CLARITY
Required processing time (days)	6	11-12	15-20	15-23	28
Subcategory of clearing methods	Organic solvent based	Organic solvent based	Organic solvent based	Aqueous solvent based	Hydrogel embedding
Fluorescent protein preservation	>1.5 years	> 4 weeks	(>1.5 years) (brain)	Not surveyed	Not surveyed

Supplementary Figure 1.

Comparison of optical bone clearing methods for murine bones according to required processing time, subcategory of clearing methods, and fluorescent protein preservation.

Supplementary Figure 2

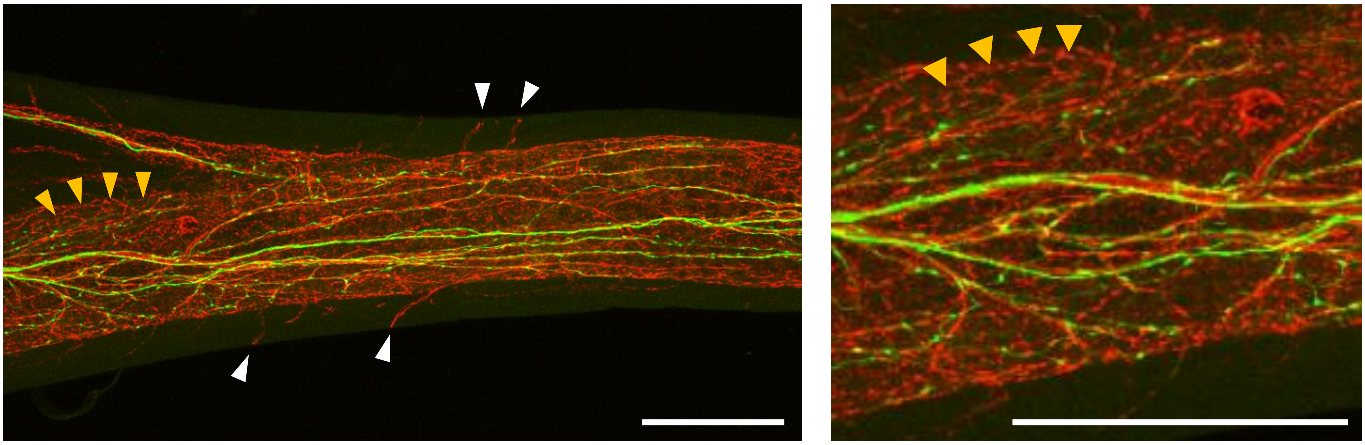

Supplementary Figure 2.

Representative Osteo-DISCO images of Sox10-positive nerves and Flt1-positive blood vessels inside the tibia from *Sox10-Venus; Flt1-tdsRed* mice. The distribution patterns of Flt1-positive blood vessels and Sox10-positive nerve fibers are not necessarily the same. Some nerve fibers, particularly thicker nerve fibers, extend alongside Flt1-positive blood vessels inside bone, but some thinner nerve fibers (orange arrowheads) do not. In addition, although nerve fibers enter the bone tissue via a specific entry site, many entry points (white arrowheads) are observed for blood vessels. Scale bars: 500 µm.

Supplementary Figure 3

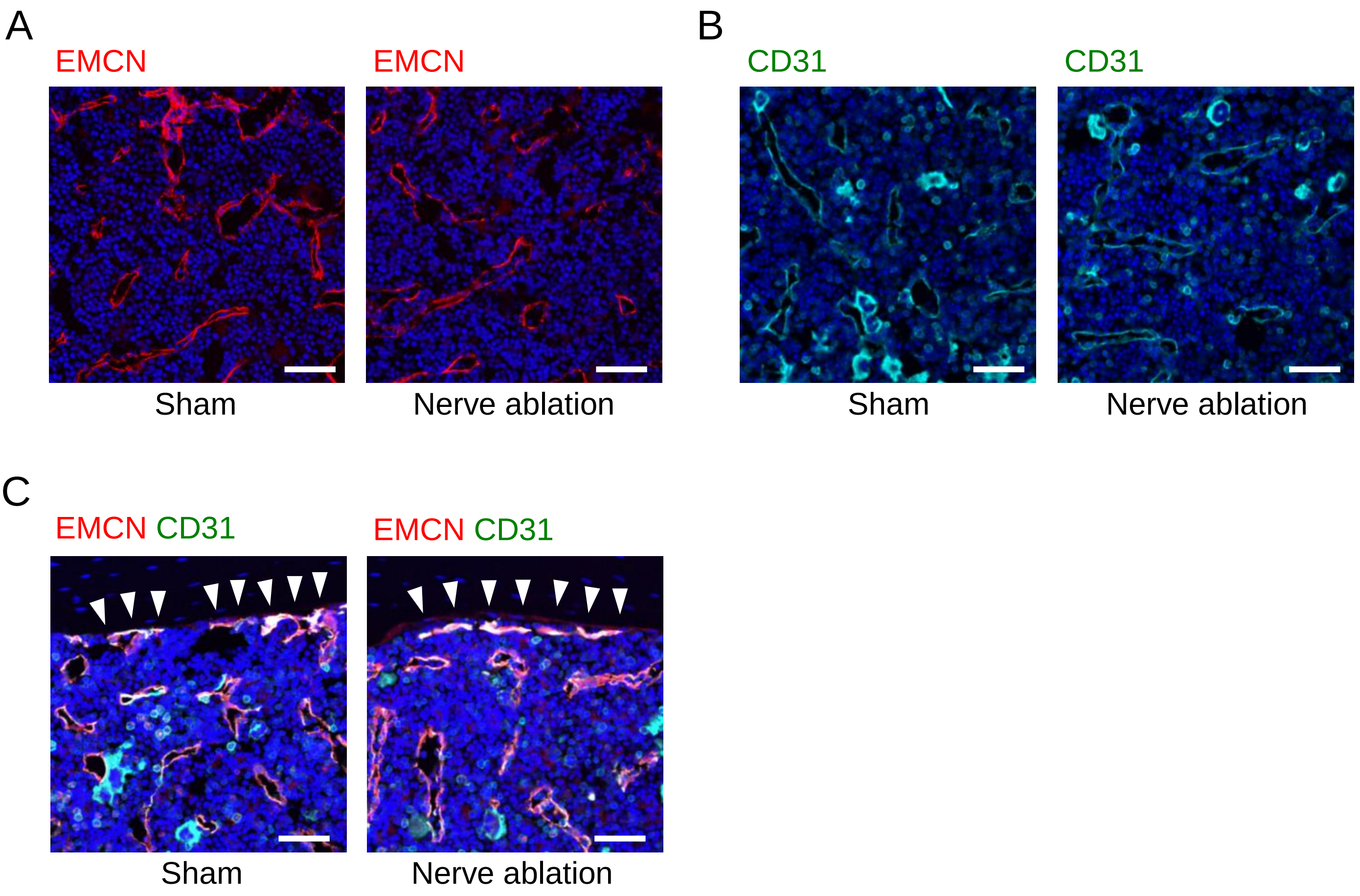

Supplementary Figure 3.

Representative images of immunofluorescence staining for EMCN (A), CD31 (B), and both EMCN and CD31 (C) in the sham and nerve ablation groups. Scale bars: 50 μm. EMCN: Endomucin. White arrowheads: Type H vessels on the endocortical surface.

Supplementary Figure 4

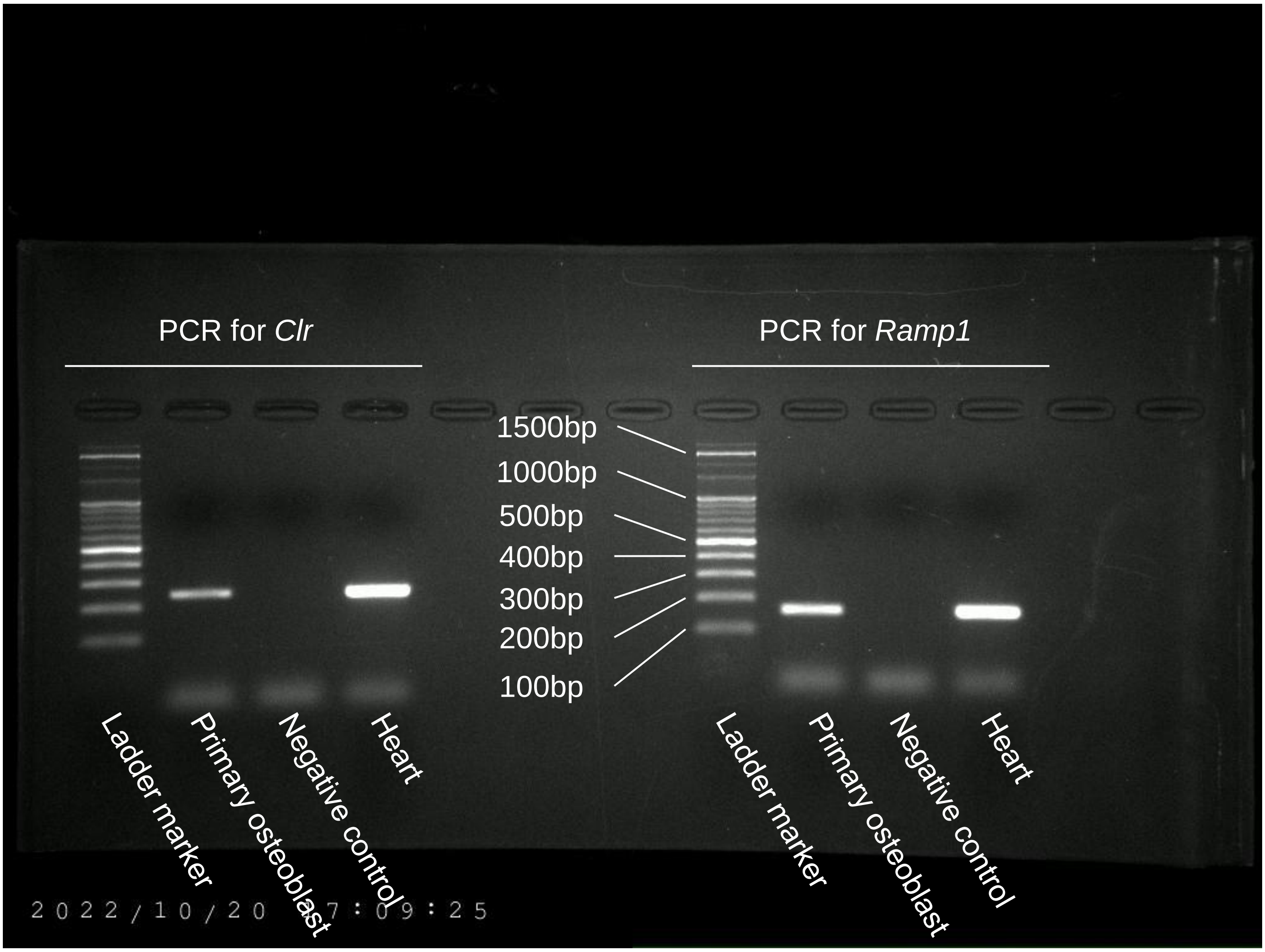

Supplementary Figure 4.

The original unprocessed image of the gel electrophoresis of PCR products. Left four lanes indicate PCR for *Clr* and right four lanes for *Ramp1*. Image sets are consisted of ladder marker, primary osteoblast sample, negative control, and murine heart sample as a positive control from left to right respectively.

Supplementary Video 1

Supplementary Video 1.

Representative 3D movie demonstrating the spatial locations of blood vessels and nerves inside tibia of a *Sox10-Venus; Flt1-tdsRed* mouse
